# Randomized control trial of prednisolone and doxycycline in patients with acute interstitial nephritis of unknown aetiology

**DOI:** 10.1186/s13063-022-07056-4

**Published:** 2023-01-05

**Authors:** Zeid Badurdeen, Neelakanthi Ratnatunga, Tilak Abeysekera, Abdul. W. M. Wazil, Premil N. Rajakrishna, Jalitha P. Thinnarachchi, Dulani D. Welagedera, Nadeeka Ratnayake, Adambarage. P. D. Alwis, Hemalika Abeysundara, Ranjith Kumarasiri, Richard Taylor, Nishantha Nanayakkara

**Affiliations:** 1 Centre for Education Research and Training On Kidney Diseases (CERTKiD), Faculty of Medicine, University of Peradeniya, Kandy, Sri Lanka; 2 Department of Pathology, Faculty of Medicine, University of Peradeniya, Kandy, Sri Lanka; 3 Renal Transplant and Dialysis Unit, Teaching Hospital, Kandy, Sri Lanka; 4 Department of Statistics and Computer Science, Faculty of Science, University of Peradeniya, Kandy, Sri Lanka; 5 Department of Community Medicine, Faculty of Medicine, University of Peradeniya, Kandy, Sri Lanka; 6 School of Public Health and Community Medicine (SPHCM), Faculty of Medicine, University of New South Wales (UNSW), Kensington, Australia

**Keywords:** Clinical trial, Prednisolone, Doxycycline, Acute interstitial nephritis, Unknown aetiology

## Abstract

**Background:**

Patients presenting with acute interstitial nephritis (AIN) of unknown aetiology, probably the earliest presentation of chronic kidney disease of unknown aetiology (CKDu), have been treated with oral prednisolone and doxycycline by physicians in Sri Lanka. This trial assessed the effectiveness of prednisolone and doxycycline based on eGFR changes at 6 months in patients with AIN of unknown aetiology.

**Method:**

A randomized clinical trial with a 2 × 2 factorial design for patients presenting with AIN of unknown aetiology (*n* = 59) was enacted to compare treatments with; A-prednisolone, B-doxycycline, C-both treatments together, and D-neither. The primary outcome was a recovery of patients’ presenting renal function to eGFR categories: 61–90 ml/min/1.73m^2^ (complete remission– CR) to 31–60 ml/min/1.73m^2^ (partial remission– PR) and 0–30 ml/min/1.73m^2^ no remission (NR) by 6 months. A secondary outcome was progression-free survival (not reaching < 30 ml/min/1.73m^2^ eGFR), by 6–36 months. Analysis was by intention to treat.

**Results:**

Seventy patients compatible with a clinical diagnosis of AIN were biopsied for eligibility; 59 AIN of unknown aetiology were enrolled, A = 15, B = 15, C = 14 and D = 15 randomly allocated to each group. Baseline characteristics were similar between groups. The number of patients with CR, PR and NR, respectively, by 6 months, in group A 3:8:2, group B 2:8:3 and group C 8:5:0 was compared with group D 8:6:1. There were no significant differences found between groups A vs. D (*p* = 0.2), B vs. D (*p* = 0.1) and C vs. D (*p* = 0.4).

In an exploratory analysis, progression-free survival in prednisolone-treated (A + C) arms was 0/29 (100%) in comparison to 25/30 (83%) in those not so treated (B + D) arms, and the log-rank test was *p* = 0.02, whereas no such difference found (*p* = 0.60) between doxycycline-treated (B + C) arms 27/29 (93%) vs those not so treated (A + D) arms 27/30 (90%).

**Conclusion:**

Prednisolone and doxycycline were not beneficial for the earliest presentation of CKDu at 6 months. However, there is a potential benefit of prednisolone on the long-term outcome of CKDu. An adequately powered steroid trial using patients reaching < 30 ml/min/1.73m^2^ eGFR by 3 years, as an outcome is warranted for AIN of unknown aetiology.

**Trial registration:**

Sri Lanka Clinical Trial Registry SLCTR/2014/007, Registered on the 31st of March 2014.

## Background

Endemic chronic kidney disease of unknown aetiology (CKDu) which is prevalent in Sri Lanka and many other tropical countries rank amongst diabetes mellitus and essential hypertension in causing chronic kidney disease (CKD) [1–5]. This condition in Sri Lanka and Nicaragua shares many demographical and clinicopathological similarities that young male agricultural workers who perform strenuous labour in hot and humid working conditions are the worst affected [6, 7]. High levels of fluoride, calcium and magnesium carbonates in drinking water may be a causative factor in Sri Lanka as would exposure to infections with Leptospira and Hantavirus, along with agrochemicals, heavy metals, ochratoxins, cyanobacterial toxins and heat stress [8–14].

CKDu is primarily a chronic interstitial disease which may have resulted from acute or subacute, low-grade recurrent interstitial nephritis [15–17]. Episodes of acute interstitial nephritis (AIN) have been clearly demonstrated in the endemic populations of Sri Lanka and Nicaragua, demographics and pathology of these nephropathies are similar [18–20]. It is thought that immunomodulatory therapy is likely to decelerate the fibrotic process and hence the severity of irreversible kidney damage [21, 22].

After leptospirosis outbreaks in the dry zone of Sri Lanka in 2008 and 2011, leptospirosis was found to be endemic in a region where CKDu is also endemic [23]. Since a high proportion of CKDu patients are farmers, there is a possibility of exposure to urine and body fluids of Leptospira-infected animals during their farming activities [11]. AIN is the main pathology of renal leptospirosis which may present in a clinical form or cause subclinical infection [24, 25]. Considering leptospirosis as a possible cause for AIN of unknown aetiology, clinicians were tending to treat with doxycycline in addition to steroids, with no substantial evidence of its effectiveness.

The objectives of the current clinical trial were to assess the effectiveness of prednisolone or doxycycline treatment, compared to those without such treatment, for patients presenting with AIN of unknown aetiology from CKDu endemic regions of Sri Lanka. Our hypothesis was that these treatments improve a patients’ presenting renal function, either to a complete remission (CR) 61–90 ml/min/1.73m^2^ eGFR or to a partial remission (PR) 60–31 ml/min/1.73m^2^ eGFR by the sixth month of intervention.

## Methods

### Trial design and patients

This study is a single-centred prospective double-blinded randomized clinical trial. This was a factorial design where two treatments were combined for evaluation in a single study. The trial was conducted at the satellite renal clinics in the North Central Regions and the Dialysis and Transplant unit of Teaching Hospital Kandy, in Sri Lanka.

AIN of unknown aetiology, an acute manifestation of CKDu which is diagnosed after exclusion of known causes for kidney diseases by history, laboratory tests and histology. Adults without any known premorbid kidney diseases from CKDu endemic regions, particularly presenting with recent onset backache, feverishness, dysuria, arthralgia and or dyspepsia were screened by serum creatinine and urinalysis. A clinical history was taken to exclude premorbid renal diseases, and recent exposure to nephrotoxins, such as over-the-counter medications non-steroidal anti-inflammatory drugs (NSAID) and proton pump inhibitors (PPI), and other treatments containing aristolochic acid. Aristolochic acid has been used in Ayurveda and traditional medicine in Sri Lanka as an ingredient of many herbal decoctions [26]. The doses of aristolochic acid preparations used in Sri Lanka are much lower than the doses associated with tubulo-interstitial nephropathy, according to Wijesinghe et al., 2016. However, the use of Aristolochic species for medicinal decoctions in Sri Lanka is considered a potential risk factor for AIN until the safe dose is defined.

Patients were diagnosed as probable AIN in the satellite renal clinics after ascertaining a few elevated serum creatinine measurements (> 116 males and > 98 µmol/L for females) and associated red cells, pus cells, and proteins in urine sediments, often within 2 weeks duration. Urine culture positives excluded. Renal ultrasonography (grayscale) was performed to confirm the presence of renal parenchymal disease, to exclude any obstructive kidney diseases, and to assess whether kidneys were not shrunken and of enough size (> 9 cm bipolar length) for biopsy. The normal sizes of kidneys for healthy individuals of CKDu endemic regions in Sri Lanka are 9.83 (1.49) cm for males and 9.46 (1.63) cm for females [27]. If consent given for biopsy and further treatment by patients, transferred to the tertiary care nephrology unit in Kandy.

**Fig. 1:**
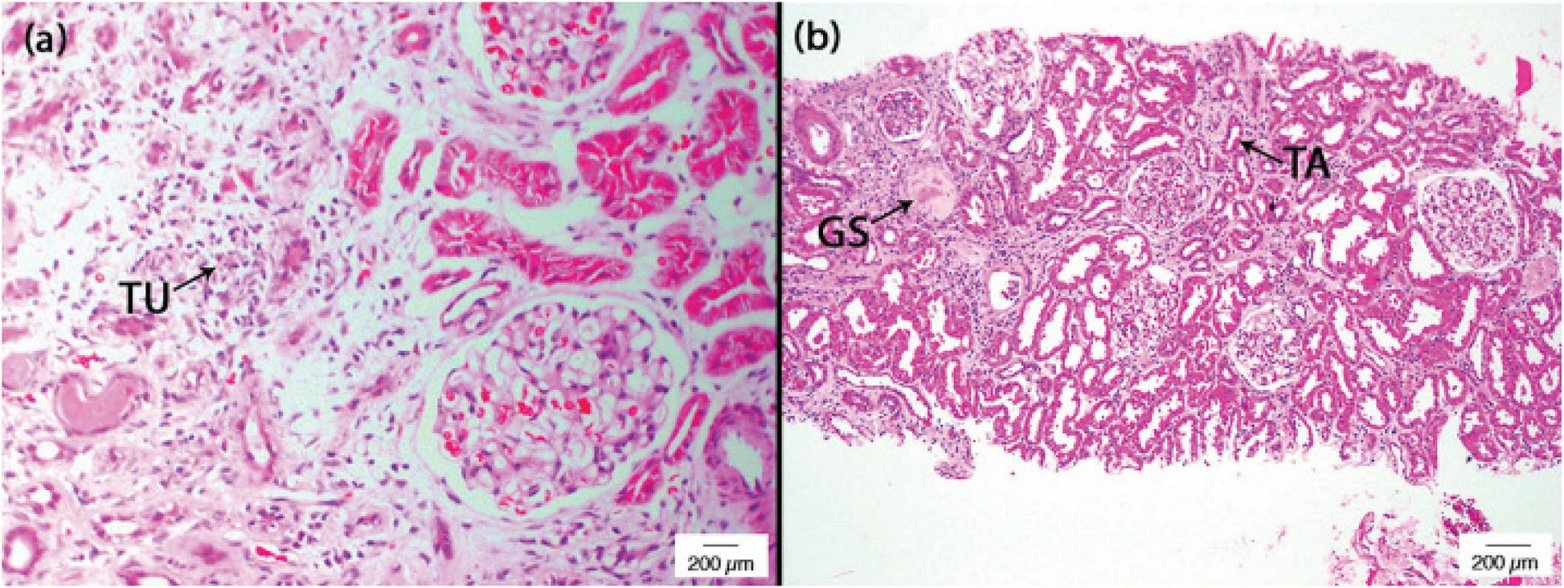
Light microscopy images of acute interstitial nephritis (AIN) from CKDu endemic regions of Sri Lanka. Histology of **a** a focus of tubulitis (TU) stained with haematoxylin and eosin (H&E); magnification × 400. **b** Sclerosed glomerulus (GS) and atrophic tubule (TA), stained with H&E; magnification × 100

Renal biopsies were examined with routine and special stains and direct immunofluorescence staining to detect immune complexes of IgG, A, and M, and complement. AIN was confirmed if there was interstitial inflammation and wide-spread tubulitis away from areas of interstitial and glomerular fibrosis, and tubular atrophy (Fig. 1). In addition, immune complex-mediated glomerular diseases, and other identifiable primary or secondary renal pathologies were excluded from histological evidence.

MAT test with a fourfold or greater rise 2 weeks apart samples have been using to diagnose cases of leptospirosis in Sri Lanka. Besides, a single reading of > 1/400 titre is supportive in clinically suspected cases when the second sample is not feasible. A titre of 1/100 or 1/200 is considered a probable case or a past infection in leptospirosis endemic regions, according to Haake and Levett et al., 2014. Cases of leptospirosis are diagnosed based on the MAT test when other serological tests and PCR amplification are not available for routine practice in low socioeconomical setups in Sri Lanka.

Biopsy-confirmed AIN cases of unknown aetiology were randomized and allocated for treatments. Serial creatinine and urinalysis were performed according to the study protocol.

### Interventions and outcomes

**Fig. 2:**
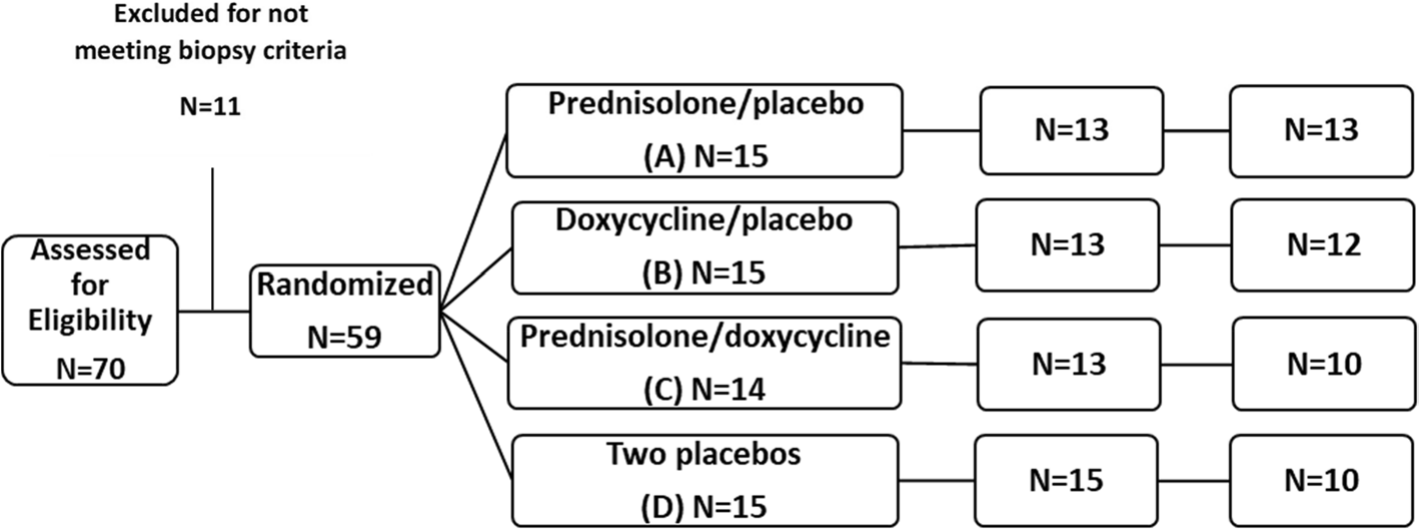
Clinical trial profile and patient flowchart

A factorial randomization was done to assign patients to one of four treatment arms (Fig. 2): arm “A” 15 patients (oral prednisolone 1 mg/kg/body weight as a single daily dose and a placebo for 1 month, tapered over 5 to 12 weeks); arm “B” 15 patients (oral doxycycline 100 mg twice day and a placebo for 1 month); arm “C” 14 patients (a combination of prednisolone and doxycycline treatments); control arm “D” 15 patients (two placebos for a period of 1 month). The treatments were commenced for all groups with a median (IQR) 20 (17–34) days after onset of symptoms.

The glomerular filtration rate (GFR) was estimated using the CKD-EPI creatinine Eq. (2009). Renal function was graded based on estimated GFR (eGFR) categories (ml/min/1.75 m^2^): stage 1 > 90, stage –2 60–89, stage –3 30–59 (stage –3a 45–59, stage –3b 30–44), and ≥ stage –4 or < 30 eGFR.

The primary outcome was a recovery of patients’ presenting renal function to eGFR categories: 61–90 ml/min/1.73m^2^ (complete remission– CR), to 31–60 ml/min/1.73m^2^ (partial remission– PR) and 0–30 ml/min/1.73m^2^ no remission (NR) by 6 months. A secondary outcome was progression-free survival (patient not reaching < 30 eGFR), between 6 and 36 months after treatments. Other secondary outcomes were remission of proteinuria by the end of 6 months of treatment and requiring renal replacement therapy (RRTs) from baseline to 36 months.

### Randomization and blinding

This is a pragmatic clinical trial conducted in the routine clinical setup. Biopsy-confirmed AIN cases of unknown aetiology were reviewed at the nephrology clinic in Kandy with their biopsy reports and explained about the clinical trial to the participants, by one of the two medical officers who were responsible for randomization and allocation of treatments. Those patients who consented for the trial were randomly allocated for treatments. The pharmacist and these two medical officers were not blinded for allocations. These staff members were not involved either in outcome evaluation or data analysis. They maintained separate trial data entry records of patients under their custody for review and re-evaluation of treatment compliance.

At the same time, trial participants and outcome adjudicators of the study had no access to trial records, thereby neither the patients nor the researchers knew who was getting which type of treatment. Furthermore, this was an individually randomized trial in which participants were randomly allocated over time and individually followed up amongst other routine clinic patients, limiting access to compare treatments. However, allocated medications were not concealed according to a double dummy technique.

### Implementation

Biopsy proved 59 cases with AIN of unknown aetiology were eligible for the trial from 70 clinically suspected cases. Eleven cases were excluded after biopsy due to eight insufficient kidney tissues for reporting, one normal kidney tissue and two glomerulonephritis. Patients were followed-up monthly at the outpatient clinic until the completion of their allocated 3 months treatments. Prednisolone was discontinued due to gastric side effects in 2 patients (group A), and 3 others (2 from group B and 1 from group C) were lost to follow-up after treatment allocation. At the end of 6 months, 54 patients (92%) completed the allocated treatments, and at the end of 36 months, 45 patients (76%) had completed their allocated treatments and were retained for follow-up of outcome (Fig. 2).

Sample size of 50 cases for each arm was calculated on the assumptions of α = 0.05; power = 0.8, and 20% effect size (to achieve a 20% reduction from 144 (± 52) µmol/L mean serum creatinine). This mean creatinine value was calculated when designing the study, from the serum creatinine values of diagnosed AIN patients in the endemic regions, recorded in the biopsy request forms. We added about 15% loss to follow-up in a trial, for a sample size of 230. However, the broad inclusion criteria of the study failed to bring the required sample size and a meaningful effect to this study at 6 months of assessment. Based on the factorial design, prednisolone-treated patients (arms A + C) and no prednisolone-treated (arms B + D) were merged for an exploratory analysis to assess the outcome by 6–36 months from recruitment which was not prespecified (Fig. 2).

### Statistical analysis

Chi-square and Fisher’s exact tests were used to compare categorical variables. Kruskal–Wallis’s test and Mann–Whitney’s test were used to compare the continuous variables. Continuous variables were expressed as medians and interquartile range (IQR). Survival analyses were performed with Kaplan–Meier curves and the group differences were estimated from the log-rank test. *P*-values < 0.05 were considered significant. Statistics were calculated using IBM SPSS version 20.

## Results

Baseline characteristics namely, age, sex, eGFR, proteinuria, MAT titre, and rest of blood and urinary inflammatory markers of all four treatment arms were similar (Table 1). Treatments were commenced for all groups at 20 (17–34) median (IQR) days after the onset of symptoms. In the primary analysis, the number of patients with complete (CR), partial (PR) and no (NR) remission of renal function by 6 months, respectively, in group A 3:8:2, group B 2:8:3 and group C 8:5:0 was compared with group D 8:5:2. There were no significant differences found between groups A vs D (*p* = 0.2), B vs D (*p* = 0.1) and C vs D (*p* = 0.4).

**Table 1:** Baseline characteristics and interventions in the clinical trial of AIN of unknown aetiology

**Description**	**Group A** **(Pred + Pla)** *N* = 15	**Group B** **(Dox + Pla)** *N* = 15	**Group C** **(Pred + Dox)** *N* = 14	**Group D** **(Pla + Pla)** *N* = 15	***P*-value**
Age median (IQR) years	44 (36–52)	47 (35–50)	48 (35–50)	47(38–52)	0.9
Number of males (%)	12 (80%)	14 (100%)	14 (93%)	15(100%)	0.1
Renal function [eGFR ml/min/1.73m^2^] median (IQR)	47 (37–52)	42 (33–52)	50 (36–55)	44 (29–52)	0.7
Proteinuria [≥ 1 + DPU cases] (%)	27	25	15	15	0.8
Hematuria (%)	33	33	0.0	22	0.2
Leukocyturia (%)	86	38	64	50	0.3
ESR (> 20 mm/h) median (IQR)	42 (23–55)	19 (15–35)	49 (30–66)	22 (14–39)	0.3
CRP—> 10 mg/L (%)	36	14	21	36	0.5
MAT titre [> 1:100 cases] (%)	33	100	33	38	0.08
MBPL, median (IQR), cm	9.6 (9.1–9.9)	9.4 (8.2–9.9)	9.6 (9.3–9.8)	9.5 (9.3–11)	0.7

*Pred* prednisolone, *Dox* doxycycline, *Pla* placebo, *IQR* interquartile range, *eGFR* estimated glomerular filtration rate, *ESR* erythrocyte sedimentation rate, *CRP* C-reactive protein, *DPU* dipstick proteinuria, *MAT* microscopic agglutination test, *MBPL* mean bipolar length of kidneys

**Fig. 3:**
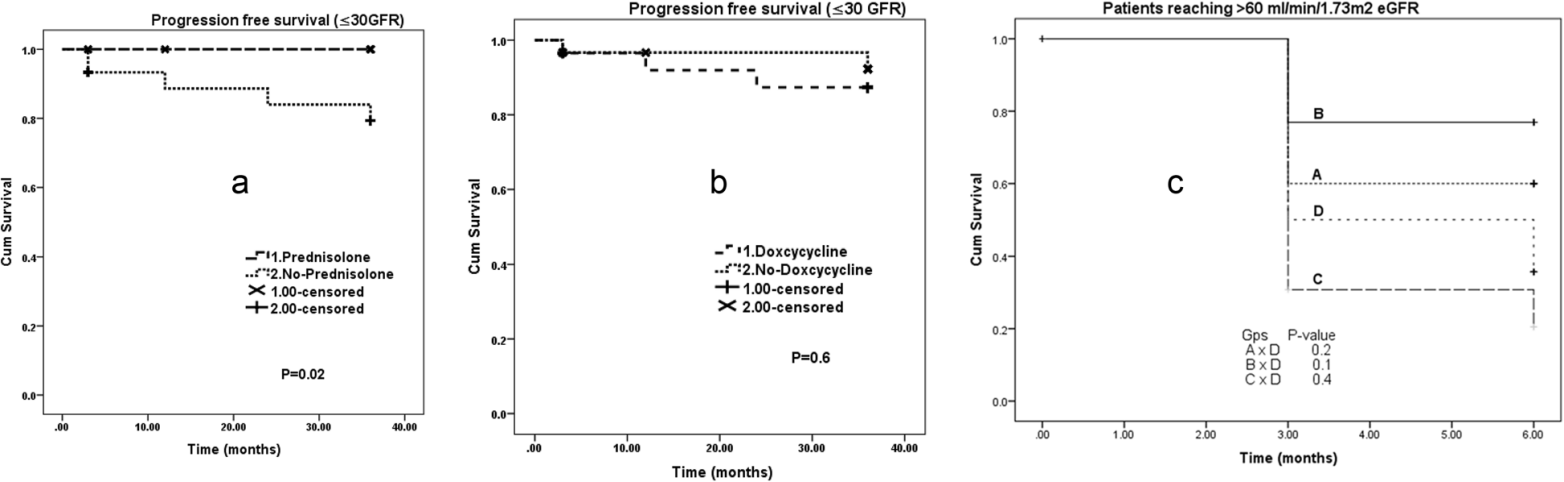
Survival plots of prednisolone and doxycycline-treated patients’ vs controls (no prednisolone or no doxycycline)

In the exploratory analysis, progression-free survival (patients not reaching < 30 ml/min/1.73m^2^ eGFR) of prednisone-treated (A + C arms) patients at 36 months was 100% while 83% for no prednisolone-treated patients (B + D arms). The log-rank test provided a *p*-value of 0.02 indicating that the difference in survival between those treatment arms was statistically significant. In contrast, progression-free survival of doxycycline-treated (B + C arms) and no-doxycycline-treated (A + D arms) was 27/29 (93%) and 27/30 (90%), respectively, the overall difference between the survival curves over 36 months was not significant (*p* = 0.62). The Kaplan Meier survival plots for exploratory analysis are depicted in Fig. 3a and b.

When changes in GFR from baseline to 6 months were adjusted for baseline GFR, there was no significant difference found in groups A vs. D (*p* = 0.87), B vs. D (*p* = 0.26) and C vs. D (*p* = 0.45).

One patient in the no prednisolone-treated (B + D arms) required renal replacement therapy by the 3^rd^ month of treatment and later underwent a kidney transplant. Proteinuria was observed only in less than 30% of AIN patients in groups at baseline. It declined over a period of 6 months from ≥ 1 + to nil: 20% of cases in group A, 25% in group B, 8% in group C and 15% in group D. Decline of proteinuria in the number of cases as a percentage between groups was not significant (*p* = 0.7). Moreover, an exploratory analysis for the primary outcome was done using patients' renal function recovery to > 61 ml/min/1.73m^2^ eGFR, a pairwise comparison done with log-rank test (Fig. 3c). In a subgroup analysis, there were five cases that progressed to > stage 4 CKD at 3 years without receiving prednisolone as a treatment option. Their baseline eGFR were also in the lower range such as 31, 42, 33, 16 and 30 ml/min/1.73m^2^. In contrast, there were three cases with baseline eGFR < 30 ml/min/m^2^ who received a course of prednisolone as an intervention. The eGFR of these three cases at 3 years improved and remained high as 50, 37 and 53 ml/min/1.73m^2^.

## Discussion

In this pragmatic clinical trial, prednisolone or doxycycline did not significantly improve renal function, 6 months after the commencement of treatment. A comprehensive understanding of the natural history of a disease is a prerequisite for designing, implementing and interpreting interventional studies. It was challenging to decide on study endpoints (development of CKD, end-stage renal disease (ESRD), renal replacement therapy (RRT) or death), duration of the study and biomarkers or surrogate end points (serum creatinine, eGFR, proteinuria) as this was the first prospective clinical trial on AIN of unknown aetiology with limited information on the natural history of the disease [28].

According to the study results, by 6 months, 55% (30/54) of trial participants have reached < 60 ml/min/1.73m^2^ eGFR, compatible with a CKD. Thus, statistically, it is a good trial outcome with a high event rate to assess interventions of CKDu progression. The event rates as ESRD, RRTs and death were extremely low (1/59) even at the end of 3 years. Considering the slow progression of CKDu, reaching < 30 ml/min/1.73m^2^ (stage 4 CKD) by 36 months was considered as a secondary outcome in this trial. The event rate for reaching < 30 ml/min/1.73m^2^ eGFR in the no prednisolone-treated patients was 5/30 (17%) by the end of 36 months. According to the subgroup analysis, steroids are beneficial even for cases with low eGFR (< stage 4 CKD) at baseline. Also, CKDu being a primary tubulointerstitial disease, a substantial proteinuria is not a feature of early disease, hence, may not be an appropriate surrogate end point for an interventional clinical trial. A tubular marker (biomarkers of tubular injury) is an optional surrogate for proteinuria [29–31].

Steroid treatment is recommended to be commenced early, at least within 7 days from initial injury for better outcomes. Nevertheless, treatments in this trial were commenced at 20 (17–34) median (IQR) days after the onset of symptoms. Until substantial knowledge is available on the time and sources of exposure and detection of suspected toxin(s) in human tissues, it is difficult to target the initial injury. According to this study, the progression in prednisolone-treated patients (none reached < 30 GFR) was relatively slower than not so treated patients (17% reached < 30GFR). Of AIN patients of Nicaragua who were followed up for 90 days after the acute onset of decline in renal function, only 21/247 (8.5%) had progressed to CKD without specific interventions [19]. In contrast, the incident stage 3 CKD at 6 months was 55% in AIN patients in our study. The significant difference in the incidence of CKD beyond months between the two acute nephropathies suggests two different etiologies. However, it is a comparison of two study groups with two different inclusion criteria.

## Conclusions

This RCT did not significantly improve the renal function of patients with AIN of unknown aetiology, up to 2 years of follow-up as designed. In the exploratory analysis, there was a slower progression to CKD stage 4 in prednisolone-treated groups. Leptospirosis was accorded as a possible cause for this AIN and adding doxycycline did not have any favourable effect on the outcome. In conclusion, empirical steroid therapy practised by physicians on AIN of unknown aetiology is a potential treatment to delay the progression of CKDu. An adequately powered steroid trial using patients reaching < 30 ml/min/1.73m^2^ eGFR by 3 years, as an outcome is warranted for AIN of unknown aetiology, in addition to surveillance for early detection and interventions.

## Data Availability

The datasets used and/or analysed during the current study are available from the corresponding author on reasonable request.

## Abbreviations

AIN: Acute interstitial nephritis
CKD: Chronic kidney disease
CKDu: Chronic kidney disease of unknown aetiology
CR: Complete remission
ESRD: End-stage renal disease
eGFR: Estimated glomerular filtration rate
IQR: Interquartile range
NR: No remission
PR: Partial remission
RRT: Renal replacement therapy
